# Dynamics of Ion Pairing in Dilute Aqueous HCl Solutions by Spectroscopic Measurements of Hydroxyl Radical Conversion into Dichloride Radical Anions

**DOI:** 10.1021/acs.jpcb.1c05642

**Published:** 2021-08-12

**Authors:** Lukasz Kazmierczak, Ireneusz Janik, Marian Wolszczak, Dorota Swiatla-Wojcik

**Affiliations:** † Institute of Applied Radiation Chemistry, Faculty of Chemistry, Lodz University of Technology, Zeromskiego 116, Lodz 90-924, Poland; ‡ Radiation Laboratory, University of Notre Dame, Notre Dame, Indiana 46556, United States; § Institute of Applied Radiation Chemistry, Faculty of Chemistry, Lodz University of Technology, Wroblewskiego 15, Lodz 93-590, Poland

## Abstract

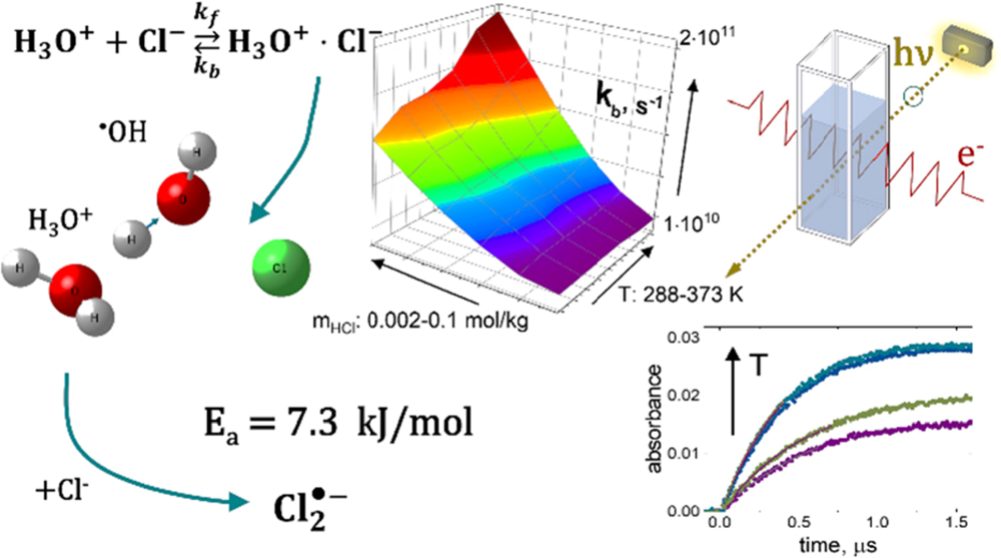

The rate of formation of dichloride anions (Cl_2_^•–^) in dilute aqueous solutions of HCl (2–100 mmol·kg^–1^) was measured by the technique of pulse radiolysis over the temperature range of 288–373 K. The obtained Arrhenius dependence shows a concentration averaged activation energy of 7.3 ± 1.8 kJ·mol^–1^, being half of that expected from the mechanism assuming the ^•^OHCl^–^ intermediate and supporting the ionic equilibrium-based mechanism, i.e., the formation of Cl_2_^•–^ in the reaction of ^•^OH with a hydronium–chloride (Cl^–^·H_3_O^+^) contact ion pair. Assuming diffusion-controlled encounter of the hydronium and chloride ions and including the effect of the ionic atmosphere, we showed that the reciprocal of τ, the lifetime of (Cl^–^·H_3_O^+^), follows an Arrhenius dependence with an activation energy of 23 ± 4 kJ·mol^–1^, independent of the acid concentration. This result indicates that the contact pair is stabilized by hydrogen bonding interaction of the solvent molecules. We also found that at a fixed temperature, τ is noticeably increased in less-concentrated solutions (*m*_HCl_ < 0.01 m). Since this concentration effect is particularly pronounced at near ambient temperatures, the increasing pair lifetime may result from the solvent cage effect enhanced by the presence of large supramolecular structures (patches) formed by continuously connected four-bonded water molecules.

## 1 Introduction

Hydrochloric acid is a strong acid. A p*K*_a_ value of −6.3 at 25 °C indicates that the concentration of undissociated molecules is negligibly small, for example, ca. 70 μM in 0.1 M in aqueous solution. However, p*K*_a_ is not enough to provide a molecular characterization of acidic aqueous solution with respect to acid concentration because the overall mechanism of acid dissociation comprises (a) the complexation of the acid molecule with a hydrogen-bonded water molecule, (b) the proton transfer from the HCl molecule to H_2_O, and (c) dissociation of the contact pair (Cl^–^.H_3_O^+^) to fully separated chloride and hydronium ions.^1^
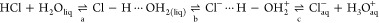
1

Equation 1 shows that the undissociated HCl molecules and the contact ion pairs (Cl^–^·H_3_O^+^) are different species, but their concentrations are connected by the sequence of equilibria (a–c). Understanding how these equilibria depend on acid concentration is important for chemical engineering, biochemistry, geochemistry, atmospheric chemistry, soil, and wastewater purification. The verification of the intermediate species in HCl acid has been studied both experimentally and theoretically using various techniques and methods, reviewed in ref (1). Recently, the structural description of medium (2.5 m) to high (16 m) concentrated solutions has been greatly improved by the combined molecular dynamics–extended X-ray absorption fine structure (MD-EXAFS) approach and state-of-the-art density functional theory (DFT) simulations.^1,2^ These studies revealed that at a higher acid concentration, almost all of the chloride ions form the contact ion pair (Cl^–^·H_3_O^+^) and that at a moderate concentration (2.5 m), the population of the contact pairs is smaller but still significant. The latter observation contrasts with the conclusion drawn from earlier Car Parrinello molecular dynamic (CPMD) simulations, indicating that in 2.7 m HCl solution, the hydronium ion forms mostly solvent separated ion pairs with Cl^–^ ions, and an abundance of the contact ion pairs in a more-concentrated solution (5.3 m).^3^ It is important to note that the radial distribution functions for Cl^–^OH_3_^+^ obtained earlier^3^ were significantly different from those obtained in more recent studies,^1^ although both studies indicated a contraction of (Cl^–^·H_3_O^+^) with the increasing acid concentration in agreement with the EXAFS data.^2^ The CPMD simulation also showed that the increase in acid concentration from 2.7 to 5.3 m extends the lifetime of the contact ion pair from *ca*. 2 to 4 ps.^3^ Except for the above-mentioned results of CPMD simulation, very little is known about the dynamics of oppositely charged ion pairs in HCl solutions and thus about the concentration dependence of equilibrium *c* in eq 1. Such knowledge is particularly lacking in the case of dilute solutions, although it has implications for the kinetic modeling of the chemistry of atmospheric, surface, waste, or chlorinated waters.^4−9^

In the present paper, we provide insight into the dynamics of such ion pairing offered by spectroscopic pulse radiolysis kinetic measurements of the hydroxyl radical (^•^OH) conversion into the dichloride radical anions (Cl_2_^•–^) in dilute aqueous solution of HCl. Pulse radiolysis provides a versatile method of generating free radicals and unstable intermediates. In irradiated aqueous solutions, the reaction of radical atom X^•^ and halide ion X^–^ produces reactive dihalide radical anions X_2_^•–^(X = Cl, Br, I), which absorb in the near UV with significant extinction coefficients. Pulse radiolysis studies at different pH values and halide-ion concentrations revealed two paths for the formation of X^•^: predominance of direct ionization of X^–^ at high halide concentration and the indirect mechanism involving the •OH radical in dilute solutions.^7,10−16^ The indirect mechanism invoking the three consecutive steps (eq 2a−2c),^12^ which accounts well for bromide and iodide solutions,^13−15,17,18^ has been recently questioned by pulse radiolysis of dilute acidic solutions of chloride ions.^7^
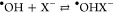
2a
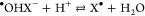
2b
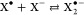
2c

The absorbance growth due to Cl_2_^•–^ observed in recent pulse radiolysis experiments^7^ was not sigmoid in shape, unlike the case of Br_2_^•–^ and I_2_^•–^,^15,17^ and the rate of dichloride ion formation was controlled by the concentration of (Cl^–^·H_3_O^+^) pairs. The role of the contact pair was confirmed by DFT computations, which showed that the ^•^OH conversion into water–dimer-stabilized Cl^•^ proceeds via fast and activationless concerted charge-proton transfer in the (H_3_O^+^ · ^•^OH · Cl^–^) complex.^7^ Irradiation of deaerated acidic solution by high-energy electron beams produces short-lived ^•^OH and H^•^ radicals.^19^ In dilute solution, diffusional encounter of ^•^OH and (Cl^–^·H_3_O^+^) initiates the formation of (H_3_O^+^ · ^•^OH · Cl^–^), and conversion of the hydroxyl radical into the chlorine atom is followed by its reaction with chloride ions to give Cl_2_^•–^. The overall mechanism is expressed by the reaction sequence (3a−3c)
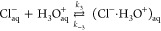
3a
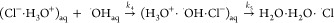
3b
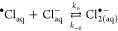
3cwhere *k*_4_ is the diffusion-controlled rate constant, and *k*_5_ ∼ 6 × 10^12^ s^–1^,^7^*k*_6_ ∼ 8.5 × 10^9^ M^–1^ s^–1^, and *k*_–6_ ∼ 6 × 10^4^ s^–1^ under ambient conditions.^20^ Thus, the technique of pulse radiolysis can be used to provide insight into the ionic equilibrium (3a) by tracing the absorbance growth of Cl_2_^•–^.

This paper reports, for the first time, the temperature dependence for the kinetics of ^•^OH conversion into Cl_2_^•–^ in irradiated HCl aqueous solutions (2 m ≤ *m*_HCl_ ≤ 0.1 m). Since persistence of the contact ion pair is inherent in the mechanistic model (reactions 3a−3c), the kinetic data presented here are used to provide insight into the dynamics of ion pairing. The lifetime of the (Cl^–^·H_3_O^+^) contact pair is discussed with respect to its dependence on acid concentration and temperature.

## 2 Methods

Hydroxyl radicals were generated by pulse radiolysis of aqueous solutions containing from 0.002 to 0.1 mol·kg^–1^ HCl. Solutions were prepared at ambient temperature using 0.1 and 1 M HCl stock aqueous solution, purchased from Sigma-Aldrich. Before irradiation, solutions were deoxygenated by purging with high purity N_2_ or Ar. The pulse radiolysis measurements were performed at the Institute of Applied Radiation Chemistry (IARC) in Lodz using 17 and 7 ns pulses from the 6 MeV ELU-6 linear accelerator, as described earlier^7^ and at the Notre Dame Radiation Laboratory (NDRL) using 1–15 ns pulses from the Titan-Beta 8 MeV pulsed electron LINAC.^21^ The dose per pulse was 55–60 Gy (17 ns) and 16–18 Gy (7 ns) at IARC and 2–30 Gy at NDRL, as measured at room temperature using N_2_O-saturated 0.01 M solution of potassium thiocyanate with Gε[(SCN)_2_^•–^] at 475 nm taken to be 5.28 × 10^–4^ m^2^ J^–1^.^22^ The absorbance growth of Cl_2_^•–^ was monitored at 340 nm, where the molar absorption coefficient ε_340_(Cl_2_^•–^) has a maximum value of ca. 9600 M^–1^ cm^–1^.^23^ The optical path length of the pulse radiolysis cell was 1 cm. The measurements were carried out for the temperature varying from 288 to 343 K using a quartz cell and within 303–373 K using a titanium cell. In the temperature range of 303–373 K, no corrosive effect on the titanium cell was observed for solutions containing at least 0.005 mol·kg^–1^ HCl.

To ensure that at 340 nm, only Cl_2_^•–^ gives rise to the absorption, we estimated possible contributions from the intermediates: ClOH^•–^, Cl^•^, and H_3_O^+^·^•^OH·Cl^–^. Taking ε_340_(ClOH^•–^) = 3000 M^–1^ cm^–1^ and the equilibrium constant for reaction 2a of *ca*. 0.7 M^–1^,^12^ the estimated absorbance of ClOH^•–^ is 3.9 × 10^–3^ at the highest concentrations of Cl^–^ and ^•^OH, i.e., 0.1 and 1.8 × 10^–5^ M (at dose 60 Gy), respectively, whereas the absorbance of Cl_2_^•–^ is at a level of 0.173. Assuming the same conditions, ε_340_(Cl^•^) = 3800 M^–1^ cm^–1^ and an equilibrium constant of 1.4 × 10^5^ M^–1^ for reaction 3c,^20^ the expected absorbance of Cl^•^ is 6.4 × 10^–6^. To assess contribution of H_3_O^+^·^•^OH·Cl^–^, we performed TD-DFT calculations of the UV–vis spectrum for two representative configurations (before and after the concerted proton-electron transfer), which were distinguished in our earlier work.^7^ The calculated extinction coefficient at 340 nm was less than 900 M^–1^ cm^–1^ (see the Supporting Information). The above estimations confirm that Cl_2_^•–^ is the only absorbing species at 340 nm.

## 3 Results

### 3.1 Temperature Dependence of Absorbance Growth

**Figure 1:**
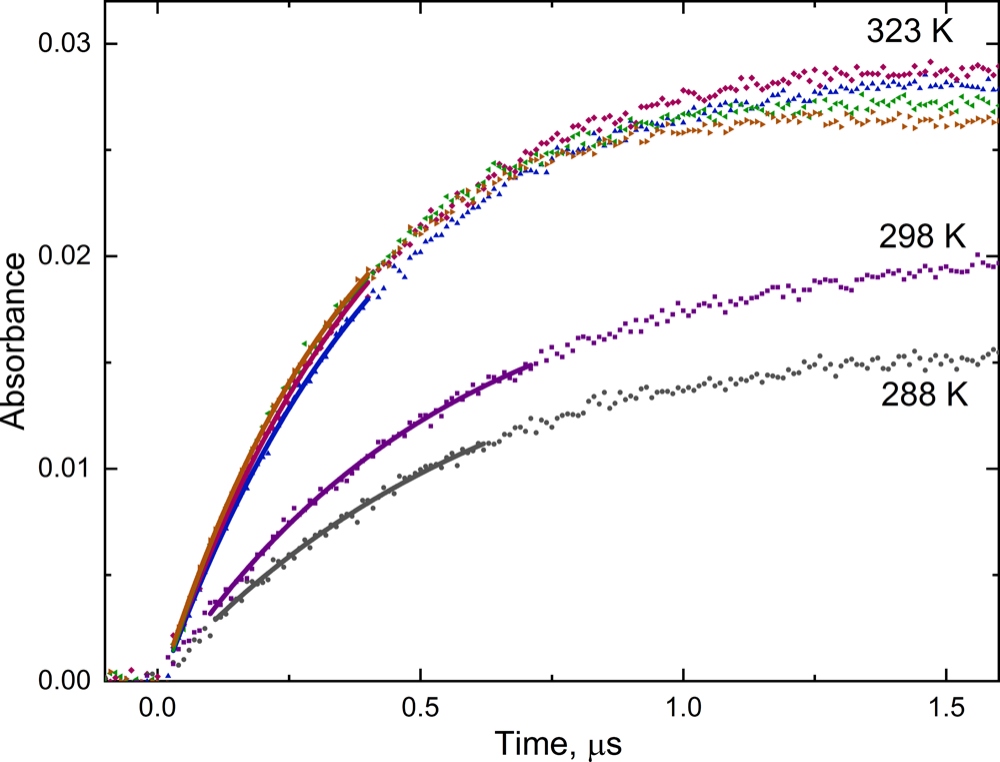
Growth of absorbance at 340 nm observed following pulse radiolysis of 0.01 m HCl aqueous solution with respect to temperature (dose 7–9 Gy). Parts of the kinetic traces fitted to the first-order growth are marked by solid lines.

Examples of traces showing the influence of temperature on the growth of absorbance at 340 nm are presented in Figure 1.

The initial parts of the kinetic traces (see solid lines in Figure 1) were fitted to the first-order growth using the Levenberg–Marquardt iteration algorithm, implemented in Origin 2019 software. The time interval for the fitting procedure was assumed in order to reduce the impact of reactions 4−9 and ensure the first-order formation of Cl_2_^•–^ (see below). The fitted first-order rate constants *k*_obs_ are collected in Table S1 (see the Supporting Information). The influence of dose on *k*_obs_ was not observed for HCl concentrations exceeding 0.03 mol·kg^–1^. For less-concentrated solutions, the growth of absorbance was noticeably affected by the decay of Cl_2_^•–^ in reaction 8, giving a larger value of *k*_obs_. Therefore, for very diluted solutions, only the kinetic traces obtained at low doses were analyzed.

### 3.2 Kinetic Model

In irradiated acidic solution, the radiation chemical yields of H^•^ and ^•^OH are comparable.^19^ Therefore, apart from reactions 3a−3c, the full kinetic model also includes reactions 4−9.
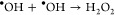
4
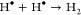
5
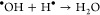
6
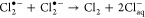
7
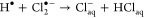
8
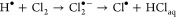
9

Numerical simulation showed particularly significant contribution of reactions 5, 6, and 8.

**Figure 2:**
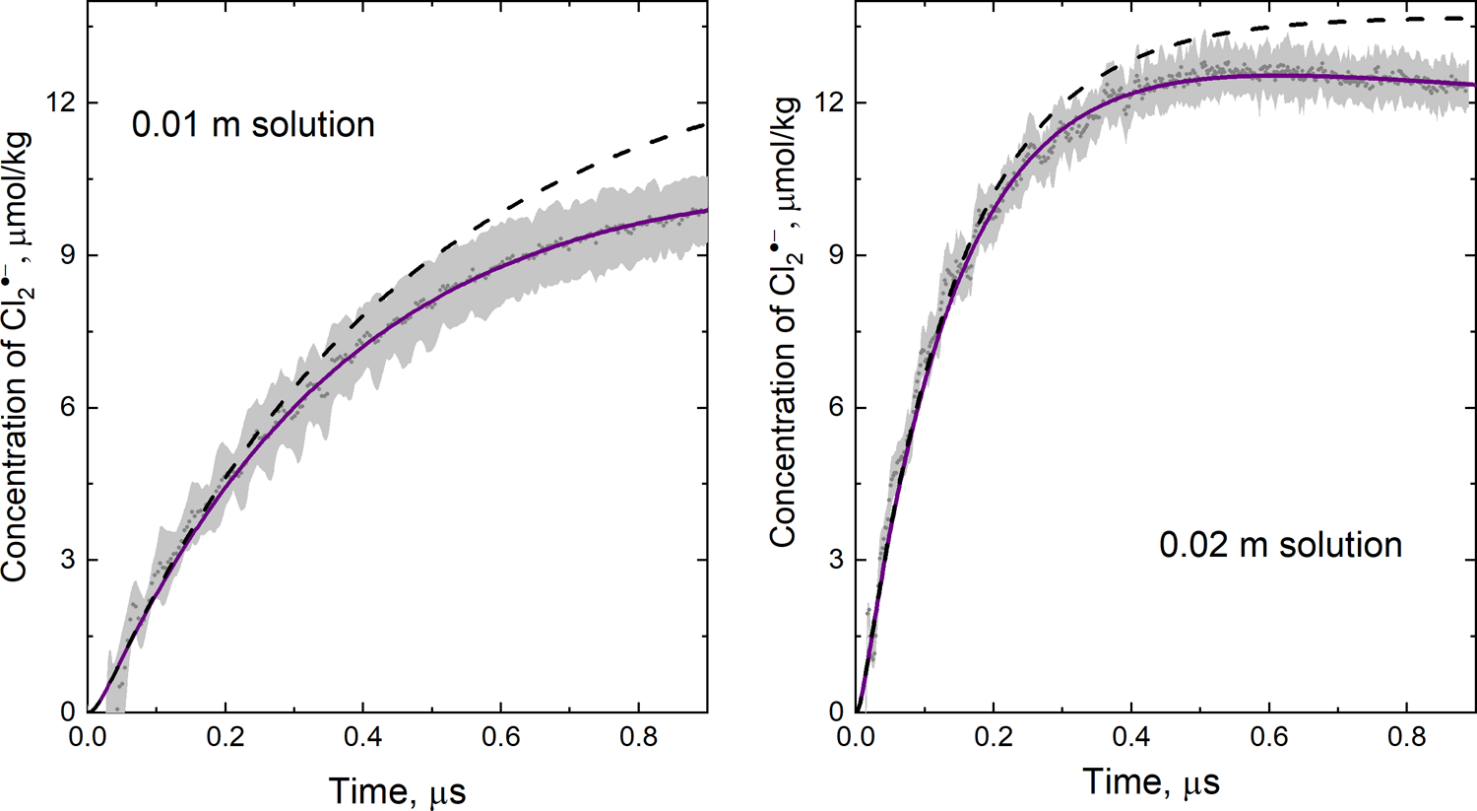
Rate of Cl_2_^•–^ formation in irradiated 0.01 and 0.02 m HCl aqueous solution at ambient temperature simulated using FACSIMILE 4 software: (solid line) – taking into account reactions 3−9, (dashed) – neglecting reactions 4−9. The rate constants for reactions 4−9 were respectively taken from refs (19) (reactions 4−6), (24) (reaction 7), and (25) (reactions 8 and 9). Experimental points refer to the dose 55–60 Gy and are shown along with the area of uncertainty.

The impact of these radical–radical reactions on the rate of Cl_2_^•–^ formation increases with the applied dose but decreases with the increasing concentration of HCl. In Figure 2, the kinetic traces simulated taking into account reactions 3−9 and neglecting radical–radical reactions 4−9 are compared with the experimental data obtained at the highest dose applied.

It is seen that despite the relatively high dose, at short time (of ca. 0.5 μs), all simulated curves are within the experimental uncertainty. The impact of reactions 5, 6, and 8 is observed at a longer time, but the discrepancy between the simulated traces is clearly smaller with a higher acid concentration.

Omitting reactions 4−9 greatly simplifies the kinetic scheme to the reaction sequence (3a−3c). Further simplifications can be made if one takes into account that *k*_5_ > > *k*_4_, and *k*_6_ × [Cl^–^] > > *k*_*–*6_ with 2 mM ≤ [HCl] ≤ 0.1 M. Using the above simplifications and assuming that equilibrium (reaction 3a) is not greatly influenced by the decay of (Cl^–^ H_3_O^+^) in reactive encounter with ^•^OH, the rate of the formation of Cl_2_^•–^ can be expressed by eq 10 (see the Supporting Information):
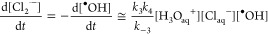
10

Since [HCl] > > [^•^OH], the conversion of ^•^OH into the Cl_2_^•–^ follows pseudo-first order kinetics with the acid concentration dependent rate constant
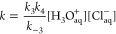
11where the rate constants and molar concentration of ions depend on temperature. The latter dependence was calculated using the polynomial expression for the density of aqueous HCl solution^26^ (see the Supporting Information for details).

### 3.3 Dynamics of Ion Pairing

Since 1/*k*_–3_ represents the lifetime of (Cl^–^·H_3_O^+^), measurements of *k* can be used to unravel the dependence of the dynamics of ion pairing on temperature and acid concentration if only *k*_3_ and *k*_4_ are known.

Taking the formation of the ionic pair as diffusion-controlled encounter of two ions, the temperature dependence for the forward rate constant of reaction 3a at negligibly small ionic strength (*I* ∼ 0) can be modeled by the Smoluchowski equation:
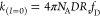
12where *N*_A_ is the Avogadro’s number, *D* is the sum of diffusion coefficients of the reactants, *R*_r_ is the reaction distance, and *f*_D_ denotes the Debye factor *f*_D_ = δ/(e^δ^ – 1), which deviates from unity when both reactants are ions. In the case of H_3_O^+^ and Cl^–^, δ = – *e*^2^/(4*π*ε_0_ε*R*_r_*k*_B_*T*) represents the ratio of the electrostatic interaction energy in a medium of relative permittivity ε at the encounter distance *R*_r_ to the thermal energy *k*_B_T. The values of ε calculated for all the solutions investigated here are given in the Supporting Information.

Since the reaction between ions occurs in an electrolyte solution of a non-negligible ionic strength, *k*_(*I* = 0)_ has to be multiplied by the square of the mean activity coefficient γ_±_^2^, being a correction of the reaction rate constant due to the presence of the ionic atmosphere.^27^ To calculate the dependence of γ_±_ on temperature and acid concentration, we selected the two most common Debye–Hückel or Pitzer–Hückel expansions for 1:1 electrolyte and used formulae for expansion coefficients provided by Partanen et al.,^28^ Saluja et al.,^29^ and Holmes et al.^30^ (see the Supporting Information). For the HCl concentration and temperature ranges of interest, here all the formulae give the values of γ_±_ that differ by less than 2%. Table S5 in the Supporting Information presents the arithmetic mean of the calculated values.

**Figure 3:**
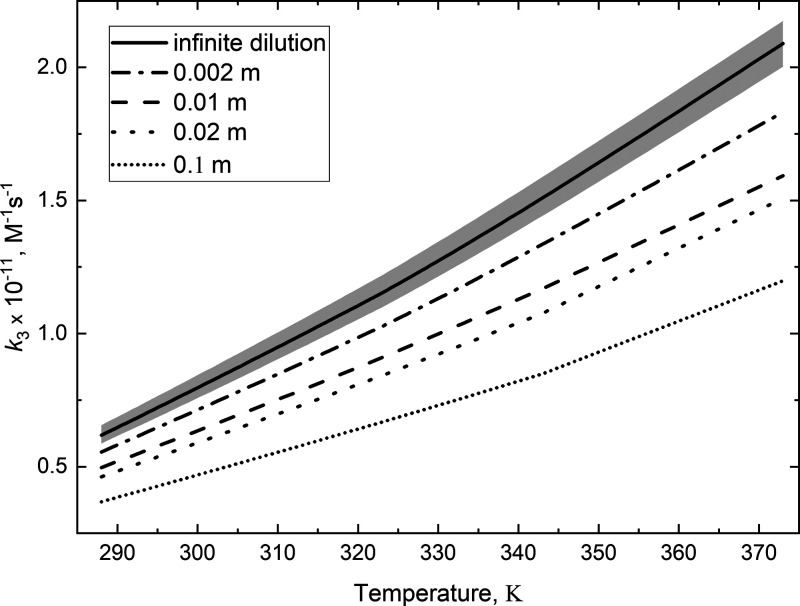
Temperature dependence for the diffusion-controlled rate of (Cl^–^·H_3_O^+^) formation in aqueous solution from infinite dilution (top solid line) to 0.1 mol·kg^–1^ (bottom dotted line). The gray area shows the range of variability resulting from the assumed encounter distance *R*_r_.

The influence of acid concentration on the diffusion-controlled rate of (Cl^–^·H_3_O^+^) formation is shown in Figure 3.

The gray area was obtained from eq 12 using the temperature dependence for the diffusion coefficient of H_3_O^+^ and Cl^–^ ions from ref (31) and two values of *R*_r_, 0.315 and 0.43 nm, respectively, denoting the lower and the upper limit for *k*_3(*I* = 0)_.

The temperature dependence for the diffusion-controlled first step of reaction sequence (3b) was obtained from eq 12 with *f*_D_ set to one. An encounter distance of 0.31 nm was assumed based on quantum chemical calculations.^7^ A room-temperature value of 2.3·10^–9^ m^2^ s^–1^ was assumed for the diffusion coefficient of both ^•^OH and (Cl^–^·H_3_O^+^) and was varied with temperature as the self-diffusion of water.^32^

## 4 Discussion

### 4.1 Activation Energy for ^•^OH Conversion into Cl_2_^•–^

Rearrangement of eq 11 gives eq 13, where the term in parentheses describes the thermodynamic equilibrium between separated H_3_O^+^ and Cl^–^ ions and the contact pairs (Cl^–^·H_3_O^+^) in solution.
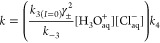
13

Equation 13 is fully consistent with the ionic-equilibrium-based mechanism proposed previously^7^ to reproduce the kinetics of Cl_2_^•–^ formation in pulse irradiated diluted solutions containing H_3_O^+^ and Cl^–^ ions coming from different electrolytes. In the range 288–373 K (15–100 °C), the temperature dependence of both *k*_3(*I* = 0)_ and *k*_4_ follows the Arrhenius dependence with activation energies of 12.7 and 16.6 kJ·mol^–1^, respectively, whereas γ_±_[H_3_O^+^] = γ_±_[Cl^–^] = γ_±_[HCl] depends linearly on the absolute temperature. The slope and intercept of the temperature dependence γ_±_ [HCl] = *a*·*T* + *b* are given in Table 1.

**Table 1:** Slope *a* and Intercept *b* of the Linear Dependence γ_±_ [HCl] = *a*·*T* + *b* Obtained Using Solution Densities and Activity Coefficients γ_±_ from Tables S3 and S5 (Supporting Information)^a^

*m*_HCl_ (mol·kg^–1^)	0.002	0.005	0.01	0.015	0.02	0.03	0.04	0.05	0.1
*a* (μM·K^–1^)	–1.30	–3.14	–7.06	–12.19	–18.36	–33.01	–49.77	–67.88	–168.14
*b* (mM)	2.29	5.58	11.15	16.96	22.98	35.51	48.46	61.67	129.22

a Coefficient of determination *R*^2^ > 0.98.

Replacing the product γ_±_^2^[H_3_O_aq_^+^][Cl_aq_^–^] by γ_±_^2^[HCl]^2^ = (*aT* + *b*)^2^, the logarithmic form of eq 13 is
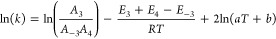
14where *A_i_* and *E_i_* denote the pre-exponential factor and the activation energy for *k*_3_, *k*_*–*3,_ and *k*_4_. The last term in eq 14 indicates some deviations from the Arrhenius dependence since the product (γ_±_[HCl]) decreases with the increasing temperature. The influence of temperature is more noticeable at increasing acid concentration.

The Arrhenius parameters for *k*_obs_ and *k*_obs_/(γ_±_[HCl])^2^ resulting from the satisfactory fits (coefficient of determination *R*^2^ > 0.81) are presented in Table 2. As expected from eq 14, the fit to the Arrhenius dependence was better in the case of *k*_obs_/(γ_±_[HCl]).^2^

**Table 2:** Arrhenius Parameters for the Observed Pseudo-First Order Rate Constants *k*_obs_ of ^•^OH Conversion into Cl_2_^•–^ and for *k*_obs_/(γ_±_[HCl])^2^ Obtained Using Weighted Linear Regression To Account for the Experimental Uncertainty^a^

	*k*_obs_		*k*_obs_/(γ_±_[HCl])^2^	
*m*_HCl_ (mol·kg^–1^)	*A* (s^–1^)	*E*_a_ (kJ·mol^–1^)	*A* (M^–2^·s^–1^)	*E*_a_ (kJ·mol^–1^)
0.005^b^	1.35 *×* 10^7^	7.4 ± 1.8	9.3 *×* 10^11^	8.4 ± 1.8
0.01^c^	2.78 *×* 10^7^	6.7 ± 1.0	5.6 *×* 10^11^	8.0 ± 0.9
0.03^d^	1.68 *×* 10^9^	11 ± 2	5.7 *×* 10^12^	13 ± 2
0.05^b^	1.80 *×* 10^8^	3.9 ± 1.1	3.1 *×* 10^11^	6.5 ± 0.9
0.1^b^			4.6 *×* 10^11^	7.9 ± 1.7
Av.^e^		7.3 ± 1.8		8.8 ± 1.8

a The satisfactory weighted linear regression fits (0.81 < *R*^2^ < 0.93) are only shown.

b Measured over 288–343 K.

c Measured over 288–373 K.

d Measured over 298–333 K.

e Averaged over the concentration range.

Within the statistical uncertainty, the activation energy for *k*_obs_/(γ_±_[HCl])^2^ does not show noticeable concentration dependence. The concentration averaged value of 8.8 ± 1.8 kJ·mol^–1^ is slightly higher than 7.3 ± 1.8 kJ·mol^–1^ obtained for *k*_obs_. As we argue below, such a result indicates that the overall process of ^•^OH conversion into Cl_2_^•–^ is not diffusion controlled.

Equation 13 would be the same as *k* = *k*_2a_*k*_2b_(*a*_H^+^_)(*a*_Cl^–^_)/(*k*_–2a_ + *k*_2b_*a*_H^+^_), earlier proposed by Jayson et al.,^12^ if *k*_–2a_ > > *k*_2b_ *a*_H^+^_, where *a*_H^+^_ and *a*_Cl^–^_ denote the activities of ions, *k*_2a_ and *k*_–2a_ are the rate constants for the forward and reverse reaction 2a, respectively, and *k*_2b_ is the rate constant of reaction 2b. Although in our experiments, *a*_H^+^_ was sufficiently small to fulfill this condition, the room-temperature values *k*_2a_ = 4.3 × 10^9^ M^–1^ s^–1^, *k*_–2a_ = 6.1 × 10^9^ s^–1^, and *k*_2b_ = 2.1 × 10^10^ M^–1^ s^–1^ given by Jayson et al.^12^ yield *k*–values about half the *k*_obs_ measured here (see the Supporting Information, Table S1), e.g., 3.14 × 10^5^ s^–1^ compared to (6.9 ± 0.2) × 10^5^ s^–1^ for 5 mmol·kg^–1^ solution. The mechanism based on reactions 2a and 2b indicates a sigmoid shape of the absorbance growth of Cl_2_^•–^, which was observed neither here (see Figure 1) nor in our earlier experiments.^7^ Dissimilarity of the chloride system compared to the bromide and iodide ones was also indicated by Yamaguchi,^33^ who demonstrated that the formation of ^•^OHCl^–^ in reaction 2a is endothermic in contrast to the exothermic formation of ^•^OHBr^–^ and ^•^OHI^–^. Moreover, the formation of Cl^•^ in reaction 2b was questioned by several computational studies strongly suggesting the importance of the contribution from a water molecule in the overall process, as discussed in detail in ref (7). Since the forward reaction 2a is endothermic,^33^ the increase in temperature shifts the equilibrium to the right. This means that at the concentration of Cl^–^ sufficiently high to neglect the backward reaction 2c, the overall process of ^•^OH conversion into Cl_2_^•–^ is controlled by diffusion. Therefore, one may expect the activation energy close to that observed for the self-diffusion of water, being equal to 16.2 kJ·mol^–1^ over the temperature range of 293–383 K.^32^ However, the temperature dependence obtained here (see Table 2) indicates that the activation energy is nearly half that. This is consistent with the mechanism based on equilibrium (3a) and at the same time indicates that ^•^OH conversion into Cl_2_^•–^ is controlled by the dynamics of ion pairing.

### 4.2 Dynamics of Ion Pairing: Effect of Temperature and HCl Concentration

**Figure 4:**
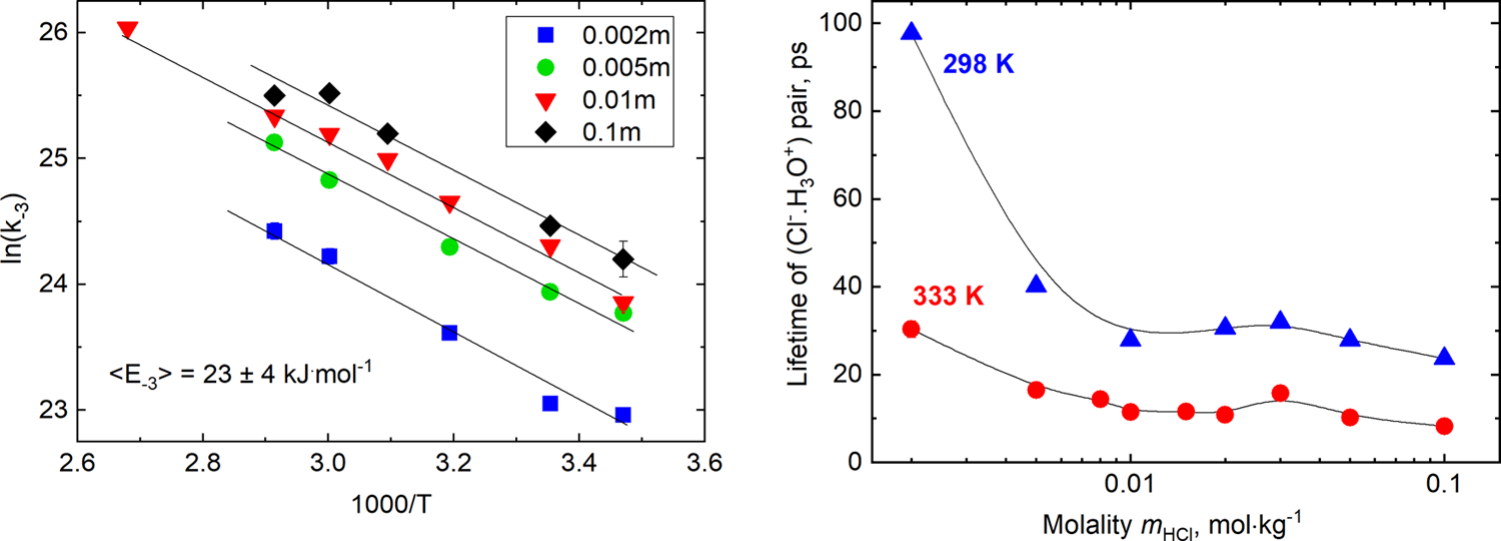
Arrhenius plots for the dependence of the rate constant of contact pair dissociation (*k*_–3_) on acid concentration, given in mol·kg^–1^ (left). Lifetime of the (Cl^–^·H_3_O^+^) pair as a function of solution molality shown for 298 and 333 K (right).

According to the DFT calculations,^7^ the concerted proton–electron charge transfer occurs when ^•^OH approaches the contact pair (Cl^–^·H_3_O^+^). The existence of (Cl^–^·H_3_O^+^) in aqueous HCl solutions was confirmed by many experimental and computational studies, using neutron and X-ray diffraction, MD-EXFAS, DFT, and CPMD techniques (see ref (1) for a review). Taking into account the structural and dynamical properties of the chloride ion and excess proton, the formation of (Cl^–^·H_3_O^+^) is not surprising. The classical MD simulation of aqueous 1.1 M NaCl solution showed that the solvation shell of Cl^–^ in water is structure-less and consists of 6–7 H_2_O molecules, preferring the OH bond oriented toward the anion.^34^ Despite hydrogen bonding interaction with the surrounding water molecules, the solvation shell is rather flexible and the influence of Cl^–^ on the motion of water molecules is rather weak, suggesting that a chloride ion may replace a water molecule without any significant distortion of the hydrogen-bond network.^35^ On the other hand, the high mobility of excess protons in water at ambient and elevated temperatures is intimately connected with the hydrogen-bond network, facilitating proton transfer from H_3_O^+^ to a neighboring hydrogen-bonded H_2_O molecule.^27^ The contact pair (Cl^–^·H_3_O^+^) may be formed when a proton transfers to one of the molecules solvating the chloride anion. This qualitative explanation is supported by DFT-based simulations.^1,3^ In our kinetic approach, the formation of (Cl^–^ H_3_O^+^) is modeled as the diffusion-controlled encounter of the two species at a distance *R*_r_ taken to be equal to 0.315 or 0.43 nm. We made this assumption guided by the position of the first and second maximum of the Cl^–^OH_3_^+^ radial distribution function reported from CPMD simulation of 2.5 m solution.^3^ The uncertainty in *k*_3(*I* = 0)_ resulting from the two values of *R*_r_ is less than 7% (see the gray area in Figure 3). Substituting the arithmetic mean of *k*_3(*I* = 0)_ in eq 13, we calculated effect of temperature and acid concentration on *k*_–3_. Good fits (*R*^2^ > 0.94) to the Arrhenius dependence were obtained for all the systems studied. Selected Arrhenius plots are shown in Figure 4 (left).

Within the uncertainty, the activation energy *E*_–3_ for the contact pair dissociation is independent of acid concentration. In the temperature range (288–373 K), the concentration averaged value of the activation energy is < *E*_–3_ > = 23 ± 4 kJ·mol^–1^, in good agreement with the value calculated from eq 14. Namely, the activation energy for *k*_obs_/(γ_±_[HCl])^2^ is *E*_3_ + *E*_4_ – *E*_–3_. Substituting 12.7 and 16.6 kJ·mol^–1^ for *E*_3_ and *E*_4_, respectively, and taking 8.8 ± 1.8 kJ·mol^–1^ for the overall process (see Table 2), we obtain *E*_–3_ = 20.5 ± 1.8 kJ·mol^–1^. These values compare well with the energy of hydrogen bonds in liquid water. Up to 373 K, the statistical distribution of a pair interaction energy in liquid water shows an attractive energy part with the maximum at about −19 kJ·mol^–1^ and the average −17.8 ± 0.2 kJ·mol^–1^.^36,37^ Therefore, it may indicate that the contact pair is stabilized by hydrogen bonding interaction of the solvent molecules. Increase in temperature promotes pair dissociation and shifts equilibrium (reaction 3a) to the left. The results presented in Figure 4(right) show that at a fixed temperature, the lifetime of the pair (Cl^–^·H_3_O^+^), being equal to 1/*k*_–3_, is increased in more diluted solutions (*m*_HCl_ < 0.01 m). The more pronounced the concentration effect is the lower the temperature is. At ambient temperature, more than a fourfold increase of the lifetime was noted (from 23.6 ps in 0.1 m solution to 97.6 ps at 0.002 m), whereas at 333 K, the increase of the pair lifetime is threefold (from 8.3 ps at 0.1 m to 30.3 ps at 0.002 m). All the calculated lifetimes are collected in Table S6 (see the Supporting Information). Although it can be argued that the effect of concentration on the diffusion of reactants was ignored in the calculation of 1/*k*_–3_, it seems to be not significant in the concentration range studied here. The reason for the observed increase may reflect the more crowded ionic environment at a higher acid concentration, which destabilizes the ion pairs through increased electrostatic interactions. However, taking into account that the concentration dependence is weaker at elevated temperatures, another reason is worth considering. Computational studies of molecular self-assembling revealed that at near ambient temperatures, the hydrogen-bond network of water comprises large supramolecular structures (patches) formed by continuously connected four-bonded molecules.^38^ The patches, embedded in less ordered but more dense regions, stiffen the water structure and enhance the solvent cage effect. The pairs formed in patches persist longer due to likely restoration in a solvent cage. At a higher concentration, more pairs are formed in less-rigid regions, where their lifetime is shorter. Since the size of patches decreases with the increasing temperature,^38^ the concentration dependence should be milder at elevated temperature, which is seen in Figure 4 (right).

In conclusion of this section, it is worth noting that Heuft and Meijer^3^ observed the formation and restoration of contact pairs in 2.7 m HCl solution at 332 K using CPMD simulation and estimated the lifetime of (Cl^–^·H_3_O^+^) to be around 8–9 ps and 10–12 ps. These estimations agree well with 8.3 ps found here for 0.1 m HCl solution at 333–343 K.

## 5 Conclusions

The pulse radiolysis measurements of the kinetics of ^•^OH conversion into Cl_2_^•–^ in dilute (0.002–0.1 mol·kg^–1^) HCl aqueous solutions revealed an activation energy of 7.3 ± 1.8 kJ·mol^–1^ for the temperature range 288–373 K (15–100 °C). The observed activation energy is half the value that might be expected for the investigated ranges of temperature and chloride ion concentration based on the mechanism (reactions 2a–c) assuming that ^•^OHCl^–^ is an intermediate. At the same time, the measured temperature dependence supports the alternative mechanism (reactions 3a–3c), proposed in our earlier work.^7^ According to this mechanism, the formation of Cl_2_^•–^ is initiated by diffusional encounter of ^•^OH with the contact pair (Cl^–^·H_3_O^+^) followed by fast concerted charge-proton transfer in the encounter complex to produce Cl^•^, subsequently reacting with Cl^–^ to give Cl_2_^•–^. We proved that at low absorbed dose, the rate of Cl_2_^•–^ formation is determined by the concentration of contact pairs (Cl^–^.H_3_O^+^), established in the equilibrium , and the rate of formation of the intermediate complex. Assuming that formation of (Cl^–^ · H_3_O^+^)_aq_ and (H_3_O^+^ · ^•^OH · Cl^–^)_aq_ is diffusion controlled, we calculated the temperature dependence of the rate constants using the Smoluchowski equation. To account for the effect of the ionic atmosphere on the former reaction, the rate constant at infinite dilution was multiplied by the square of the mean activity coefficient γ_±_. For calculations of γ_±_ as a function of temperature and acid concentration, we selected the Debye–Hückel and Pitzer–Hückel expansions for 1:1 electrolyte. It follows that in the studied ranges of temperature and acid concentration, the product of γ_±_ and acid concentration, expressed in M, linearly depends on temperature, resulting in some deviations from the Arrhenius dependence of the measured rate constant of Cl_2_^•–^ formation, *k*_obs_. A much better fit to the Arrhenius dependence was obtained for *k*_obs_/(γ_±_[HCl])^2^ showing the concentration averaged value of the activation energy of 8.8 ± 1.8 kJ·mol^–1^.

The second part of this work provides insight into the dynamics of ion pairing. Based on the measured kinetics of Cl_2_^•–^ formation, we calculated the rate constant, *k*_*–*3_, for back dissociation of (Cl^–^·H_3_O^+^) and its dependence on temperature and HCl concentration. Good fits to the Arrhenius dependence of *k*_*–*3_ were obtained for all the systems studied. In the temperature range (288–373 K), the activation energy for the contact pair dissociation of 23 ± 4 kJ·mol^–1^ was obtained, independent of acid concentration. This result indicates that the contact pair is stabilized by hydrogen bonding interaction of the solvent molecules. At a fixed temperature, the lifetime of the pair (Cl^–^·H_3_O^+^) is decreased in more concentrated solutions (*m*_HCl_ > 0.01 m), likely due to a more crowded ionic environment, destabilizing the ion pairs through increased electrostatic interactions. However, as this concentration effect is particularly pronounced at near ambient temperatures, the increasing pair persistence may result from the solvent cage effect enhanced by the presence of large supramolecular structures (patches) formed by continuously connected four-bonded molecules. At a higher concentration, more pairs are formed in less-rigid regions, where restoration in a solvent cage is less likely. Since the size of patches decreases with the increasing temperature, a weaker concentration dependence is observed at elevated temperature.

Finally, the present work proves that the technique of pulse radiolysis is useful for studying ionic equilibria in dilute chloride systems important for chemical engineering, biochemistry, geochemistry, atmospheric chemistry, soil, and wastewater purification.

## Supporting Information Available

The first-order rate constants of the formation of Cl_2_^•–^, kinetics of Cl_2_^•–^ formation, dependence of density of aqueous HCl solution on temperature, dependence of relative permittivity of solution on acid concentration and temperature, dependence of the mean activity coefficient on acid concentration and temperature, lifetime of the pair (Cl^–^·H3O^+^), and calculated UV–vis spectra of representative chlorine complexes (PDF)
