# Supplementary material for: Dynamics of Ion Pairing in Dilute Aqueous HCl Solutions by Spectroscopic Measurements of Hydroxyl Radical Conversion into Dichloride Radical Anions
Source: J Phys Chem B. 2021 Aug 12;125(33):9564–71. doi: 10.1021/acs.jpcb.1c05642 (PMC8404193; doi:10.1021/acs.jpcb.1c05642)
Supplement: Supplementary file 1 — jp1c05642_si_001.pdf [file jp1c05642_si_001.pdf]

Dynamics of Ion Pairing in Dilute Aqueous HCl Solutions by Spectroscopic Measurements of Hydroxyl Radical Conversion into Dichloride Radical Anion

Lukasz Kazmierczak[†], Ireneusz Janik[‡], Marian Wolszczak[††], Dorota Swiatla-Wojcik[†*]

[†] Institute of Applied Radiation Chemistry, Faculty of Chemistry,
Lodz University of Technology, Zeromskiego 116, 90-924 Lodz, Poland

[‡] Radiation Laboratory, University of Notre Dame, Notre Dame 46556, USA

[††] Institute of Applied Radiation Chemistry, Faculty of Chemistry,
Lodz University of Technology, Wroblewskiego 15, 93-590 Lodz, Poland

1. The first order rate constants of the formation of $Cl_2^{\bullet-}$.

Table S1. The first-order rate constants $k_{obs}\times10^{-6}$ fitted[a)] to the growth of absorbance measured at 340 nm, being the absorption maximum of $Cl_2^{\bullet-}$.

m_{HCl}[mol·kg^{-1}]	288.15 K	298.15 K	313.15 K	323.15 K	333.15 K	343.15 K	373.15 K
0.002	0.20 ± 0.01	0.28 ± 0.01	*0.29 ± 0.03*	-	0.30 ± 0.05	0.32 ± 0.05	-
0.005	0.52 ± 0.05	0.69 ± 0.02	0.86 ± 0.05	-	0.95 ± 0.10	0.94 ± 0.07	-
0.008	-	-	-	-	2.04 ± 0.09	3.01 ± 0.08	-
0.01	1.82 ± 0.13	1.82 ± 0.03	2.26 ± 0.19	2.24 ± 0.19	2.5 ± 0.3	2.8 ± 0.4	2.9 ± 0.5
0.015	-	-	-	-	5.4 ± 0.3	6.2 ± 0.4	-
0.02	4.8 ± 0.6	7.5 ± 0.3	*9.5 ± 0.8*	8.3 ± 0.4	8.6 ± 0.5	9.1 ± 0.5	-
0.03	-	15.7 ± 0.6	*23 ± 4*	27 ± 4	*26 ± 3*	-	-
0.04	-	35 ± 3	-	-	38 ± 2	-	-
0.05	32 ± 4	38.4 ± 1.9	-	45 ± 5	44 ± 4	44 ± 5	-
0.1	100 ± 40	119 ± 6	-	130 ± 30	125 ± 16	160 ± 20	-

[a)] Non-linear curve fitting procedure using Levenberg-Marquardt iteration algorithm employed in OriginLab 2019; k_{obs} represent average of 3 – 5 profiles; uncertainty corresponds to root mean squared error of the fitted values; the determination coefficient R^2 ranged from 0.9048 to 0.9997, except for the data in italic where $0.7840 < R^2 < 0.8843$.

2. Kinetics of $Cl_2^{\bullet-}$ formation

Taking into account the steady state approximation for $(H_3O^+ \cdot {}^{\bullet}OH \cdot Cl^-)$ in reaction (3b), and assuming $k_{-3} >> k_4[^{\bullet}OH]$, $k_6[Cl^-] >> k_{-6}$, the differential rate equations for a simplified scheme involving equilibrium (3a) and reactions (3b-c) are:

$$\frac{d[(Cl^- \cdot H_3O^+)]}{dt} = k_3[H_3O^+][Cl^-] - k_{-3}[(Cl^- \cdot H_3O^+)] \qquad (S1)$$

$$\frac{d[^{\bullet}OH]}{dt} = -k_4[(Cl^- \cdot H_3O^+)][^{\bullet}OH] \qquad (S2)$$

$$\frac{d[Cl^{\bullet}]}{dt} = k_4[(Cl^- \cdot H_3O^+)][^{\bullet}OH] - k_6[Cl^{\bullet}][Cl^-] \qquad (S3)$$

$$\frac{d[Cl_2^{\bullet-}]}{dt} = k_6[Cl^{\bullet}][Cl^-] \qquad (S4)$$

Solution of eq. (S1) is

$$[(Cl^- \cdot H_3O^+)] = \frac{k_3}{k_{-3}}[H_3O^+][Cl^-]\left(1 - e^{-k_{-3}t}\right) \qquad (S5)$$

Substituting solution of eq. (S1) into eq. (S2) one obtains:

$$\frac{d[^{\bullet}OH]}{dt} = -\frac{k_4k_3}{k_{-3}}[H_3O^+][Cl^-][^{\bullet}OH](1 - e^{-k_{-3}t}) \cong -\frac{k_4k_3}{k_{-3}}[H_3O^+][Cl^-][^{\bullet}OH] \qquad (S6)$$

Where $e^{-k_{-3}t} \cong 1$ taking into account the time span of measurements and $k_{-3} > 10^9$ s^{-1} as expected.

With the boundary condition $[^{\bullet}OH](t = 0) = c_0$, at low dose being of the order of 10^{-6} M and thus implying $[H_3O^+][Cl^-]$ = const, solution of eq. (S6) is

$$[^{\bullet}OH] = c_0e^{-\alpha t}\text{ , where } \alpha = \frac{k_4k_3[H_3O^+][Cl^-]}{k_{-3}} \qquad (S7)$$

Thus solution of eq. (S3) with the boundary condition $[Cl^{\bullet}](t = 0) = 0$ is

$$[Cl^{\bullet}] \cong c_0\frac{\alpha}{\alpha - k_6[Cl^-]}(e^{-k_6[Cl^-]t} - e^{-\alpha t}) \qquad (S8)$$

$$= \frac{\alpha}{\alpha - k_6[Cl^-]}\{ e^{-(\alpha + k_6[Cl^-])t} - 1\}[^{\bullet}OH]$$

Since k_6 and k_4 are of similar order, $k_3/k_{-3} < 1$, [HCl] ≤ 0.1 M and t < 10^{-6} s, one can neglect α as small compared to $k_6[Cl^-]$ and neglect the exponential term exp{-(α+k$_6$[Cl$^-$])t}. Then [Cl$^•$] substituted to eq. (S4) gives

$$\frac{d[Cl_2^{\bullet-}]}{dt} \cong \alpha[^\bullet OH] = -\frac{d[^\bullet OH]}{dt} \cong \frac{k_4 k_3}{k_{-3}}[H_3O^+][Cl^-][^\bullet OH] \quad (S9)$$

3. Density of aqueous HCl solution in dependence of temperature T

To describe the temperature dependence for the density ϱ of aqueous HCl solution Clegg and Wexler [S1] used the following polynomial expression:

$$\varrho(T) = \sum_{k=0}^{9} p_k c_{\%(w/w)}^k + (T - T_0)(q_1 - q_2 T_0) + q_2(T^2 - T_0^2)/2 \quad (S10)$$

where T_0 = 283.15 K, $q_1 = r_1 + r_2 c_{\%(w/w)}^{r_3}$, $q_2 = r_4 + r_5\sqrt{c_{\%(w/w)}} + r_6 c_{\%(w/w)}$, $c_{\%(w/w)}$ is the mass fraction of HCl in solution, and .$p_{k=0,..,9}$ r_1, r_1, …, r_6 are fitting parameters to the experimental data collected in Table S2. The calculated densities are given in Table S3.

Table S2. Adjustable parameters for eq. (S10) obtained in this work using OriginPro 2021 nonlinear fitting procedure.

k	r_k	p_k
0		0.99707
1	$-3.71636144 \cdot 10^{-4}$	$4.91 \cdot 10^{-3}$
2	$-2.097063856 \cdot 10^{-3}$	$-1.57213 \cdot 10^{-5}$
3	1.8829510589	$1.32583 \cdot 10^{-6}$
4	$-1.7085424103 \cdot 10^{-5}$	$-3.81277 \cdot 10^{-8}$
5	$5.1656343322 \cdot 10^{-5}$	$5.67147 \cdot 10^{-10}$
6	$-3.4570919219 \cdot 10^{-5}$	$-3.16067 \cdot 10^{-12}$
7		$-7.86698 \cdot 10^{-14}$
8		$1.16366 \cdot 10^{-15}$
9		$-4.21105 \cdot 10^{-18}$

Table S3. Density of aqueous solution of HCl given in kg·m^{-3} calculated from eq. (S10). Data for pure water given in the first row are from ref. [S2].

m_{HCl}[mol·kg^{-1}]	288.15 K	298.15 K	313.15 K	323.15 K	333.15 K	343.15 K	373.15 K
0	999.2	997.0	992.2	988.0	983.2	977.7	958.4
0.002	1001.0	997.4	995.4	991.0	985.9	980.3	974.1
0.005	1002.1	997.8	995.6	991.1	986.3	981.3	976.1
0.008	1003.2	998.2	995.7	990.6	985.6	980.4	975.3
0.01	1003.9	998.4	995.7	990.2	984.8	979.4	974.0
0.015	1005.6	998.8	995.5	988.9	982.5	976.1	969.9
0.02	1007.1	999.1	995.2	987.5	980.0	972.6	965.3
0.03	1009.6	999.6	994.7	985.0	975.4	966.0	956.8
0.04	1011.7	1000.0	994.2	982.8	971.6	960.5	949.6
0.05	1013.3	1000.2	993.8	981.0	968.4	955.9	943.6
0.1	1018.0	1000.9	992.4	975.5	958.7	942.0	925.4

4. Relative permittivity of solution in dependence of acid concentration and temperature

These dependencies were obtained using the scaling procedure proposed by Artemov *et al.* [S3] who assumed that the hypothetical electrophoretic effect results from intrinsic high concentration of hydronium cations in HCl solution:

$$\varepsilon(T) = (\Delta\varepsilon + \varepsilon_\infty) \cdot \frac{\varepsilon_{\mathrm{H_2O}}(T)}{\varepsilon_{\mathrm{H_2O(298K)}}} \qquad \text{(S11)}$$

where $\varepsilon_{\mathrm{H_2O}}$ is the relative permittivity of water, $\Delta\varepsilon$ is the concentration dependent correction resulting from the presence of HCl, and ε_∞ is the high frequency dielectric constant, assumed to be 5 at 25 °C.

Table S4. The relative permittivity of aqueous HCl solution calculated as a function of temperature and acid concentration using extrapolated data from refs. [S3,S4] and the temperature dependence for ε_{H_2O} [a)] from ref. [S5].

m_{HCl}[mol·kg^{-1}]	288.15 K	298.15 K	313.15 K	323.15 K	333.15 K	343.15 K	373.15 K
0	82.15	78.40	73.11	69.78	66.61	63.59	55.32
0.002	82.23	78.48	73.18	69.85	66.68	63.65	55.38
0.005	82.20	78.45	73.15	69.82	66.65	63.63	55.36
0.008	82.16	78.42	73.12	69.80	66.63	63.60	55.33
0.01	82.14	78.40	73.10	69.78	66.61	63.58	55.32
0.015	82.09	78.34	73.05	69.73	66.56	63.54	55.28
0.02	82.03	78.29	73.01	69.69	66.52	63.50	55.25
0.03	81.93	78.19	72.91	69.59	66.43	63.42	55.17
0.04	81.82	78.09	72.81	69.50	66.34	63.33	55.10
0.05	81.71	77.98	72.72	69.41	66.26	63.25	55.03
0.1	81.17	77.47	72.24	68.95	65.82	62.83	54.66

[a)] $\varepsilon_{H_2O}(T) = 5321 \cdot T^{-1} + 233.76 - 0.9297 \cdot T + 0.1417 \cdot 10^{-2} \cdot T^2 - 0.8292 \cdot 10^{-6} \cdot T^3$

5. Mean activity coefficient in dependence of acid concentration and temperature

Debye-Hückel and Pitzer-Hückel models of electrolyte solutions treat the solvent as a dielectric continuum. and an electrolyte as an ubiquitous solute. Under these assumptions. the activity of the electrolyte depends on the concentration and the activity coefficient $\gamma_\pm$. describing the interaction energy of ions in solution. To calculate $\gamma_\pm$ we have selected the two most commonly used expansions for 1:1 electrolyte. respectively given by eqs. (S12) and (S13).

$$\ln(\gamma_\pm) = -\frac{3A_\phi\sqrt{m}}{1+B\sqrt{m}} + h_1\frac{m}{m^\circ} + b_2\left(\frac{m}{m^\circ}\right)^2 + b_3\left(\frac{m}{m^\circ}\right)^{\frac{7}{2}} - \ln\left(1+2M_{H_2O}m\right). \tag{S12}$$

$$\ln(\gamma_\pm) = f^\gamma + B^\gamma \cdot \frac{m}{m^\circ} + \frac{3}{2}C^\phi\left(\frac{m}{m^\circ}\right)^2. \tag{S13}$$

where m is the molality, expressed in mol·kg^{-1}, $m° = 1\ \text{mol} \cdot \text{kg}^{-1}$, M_{H_2O} is the molar mass of water expressed in kg·mol^{-1}. The osmotic coefficient, $A_\phi(T) = \frac{e^3}{12\pi}\sqrt{\frac{N_A \varrho}{2(\varepsilon\varepsilon_0 k_B T)^3}}$, where e is the elementary charge, N_A is the Avogadro's number, ε_0 is the permittivity of free space, and k_B is the Boltzmann constant, should be expressed in (mol·kg^{-1})$^{-1/2}$. For HCl concentration less than 5 mol·kg^{-1} , the parameters B = 1.4 (mol·kg^{-1})$^{-1/2}$, b_2 = 6.0·10^{-3}, b_3 = -9.7·10^{-5} are temperature independent, and h_1 = 0.33866 – 1.2837·10^{-3} (T – 298.15) depends linearly on T [S6]. The formulae for $f^\gamma, B^\gamma, C^\Phi$ were derived from the calorimetric data reported by Saluja *et al.* [S7], and Holmes *et al.* [S8], and later tested by Partanen *et al.* [S6]. The activity coefficients calculated from eq. (S12) and eq. (S13) using the formulae for $f^\gamma, B^\gamma, C^\Phi$ from refs. [S6-S8], differ by less than 2 %. The values presented in Table S5 represent the arithmetic mean.

Table S5. The average activity coefficient $\gamma_\pm$ calculated for aqueous HCl solution in dependence of temperature and molality.

m_{HCl}[mol·kg^{-1}]	288.15 K	298.15 K	313.15 K	323.15 K	333.15 K	343.15 K	373.15 K
2	0.9532	0.9524	0.9510	0.9500	0.9489	0.9477	0.9438
5	0.9297	0.9285	0.9265	0.9249	0.9233	0.9215	0.9154
8	0.9143	0.9128	0.9103	0.9085	0.9064	0.9042	0.8967
10	0.9061	0.9045	0.9018	0.8997	0.8975	0.8951	0.8869
15	0.8897	0.8879	0.8848	0.8824	0.8799	0.8771	0.8675
20	0.8769	0.8750	0.8716	0.8690	0.8662	0.8632	0.8526
30	0.8574	0.8553	0.8516	0.8487	0.8456	0.8422	0.8304
40	0.8427	0.8405	0.8365	0.8335	0.8301	0.8265	0.8139
50	0.8309	0.8286	0.8245	0.8213	0.8178	0.8140	0.8007
100	0.7931	0.7906	0.7861	0.7825	0.7784	0.7740	0.7584

6. Lifetime of the pair (Cl^{-}·$H3O^{+}$)

Table S6. The lifetime of the pair (Cl^{-}·$H3O^{+}$) in ps calculated as the reciprocal of the rate constant k_{-3}.

m_{HCl}[mol·kg^{-1}]	288.15 K	298.15 K	313.15 K	323.15 K	333.15 K	343.15 K	373.15 K
0.002	106.6 ± 1.5	97.6 ± 1.2	55.6 ± 1.7	-	30.3 ± 1.9	24.8 ± 1.7	-
0.005	47.5 ± 0.7	40.1 ± 0.2	28.1 ± 0.2	-	16.5 ± 0.3	12.2 ± 0.2	-
0.008	-	-	31.1 ± 0.2	-	14.4 ± 0.1	15.0 ± 0.1	-
0.01	43.7 ± 0.5	27.8 ± 0.2	19.6 ± 0.3	14.0 ± 0.2	11.4 ± 0.2	9.9 ± 0.3	4.9 ± 0.2
0.015	-	-	-	-	11.6 ± 0.1	10.0 ± 0.1	-
0.02	30.5 ± 0.8	30.5 ± 0.3	22.2 ± 0.3	14.0 ± 0.1	10.8 ± 0.1	8.7 ± 0.1	-
0.03	-	31.9 ± 0.1	25.6 ± 0.1	21.2 ± 0.1	15.7 ± 0.3	-	-
0.05	36.3 ± 0.1	27.8 ± 0.3	-	13.9 ± 0.3	10.2 ± 0.2	8.0 ± 0.2	-
0.1	30.9 ± 4.4	23.6 ± 0.2	-	11.4 ± 0.5	8.3 ± 0.2	8.4 ± 0.2	-

7. Calculated UV-Vis spectra of representative chlorine complexes

UV-Vis spectra of two representative, optimised complexes were calculated using Gaussian 09W software and time dependent (TD) DFT method coupled with UB3LYP functional and 6-311++g(3df,3pd) basis set. The polarizable continuum model with integral equation formalism (IEFPCM) was assumed for the aqueous solvent. Six excited states were considered. The calculated spectra are presented in Figure S1.

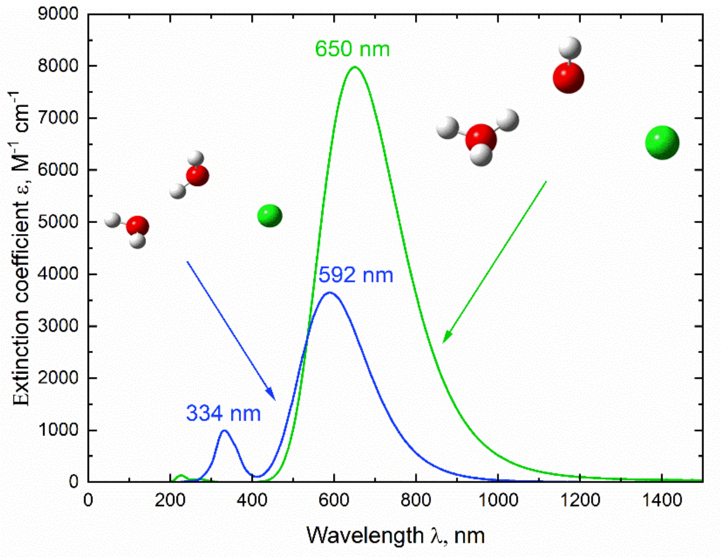

Figure S1. UV-Vis spectra of two chlorine complexes calculated using TD-DFT method: before (green) and after (blue) the concerted electron-proton transfer. Geometries of the respective complexes are also shown in the figure.

References:

[S1] Clegg. S.L.; Wexler. A.S. Densities and Apparent Molar Volumes of Atmospherically Important Electrolyte Solutions. 1. The Solutes H_2SO_4. HNO_3. HCl. Na_2SO_4. $NaNO_3$. NaCl. $(NH_4)_2SO_4$. NH_4NO_3. and NH_4Cl from 0 to 50 °C. Including Extrapolations to Very Low Temperature and to the Pure Liquid State. and $NaHSO_4$. NaOH. and NH_3 at 25 °C. *J. Phys. Chem. A* **2011**, *115*. 3393.

[S2] Wagner. W.; Pruß. A. The IAPWS formulation 1995 for the thermodynamic properties of ordinary water substance for general and scientific use. *J. Phys. Chem. Ref. Data* **2002**, *31*. 387.

[S3] Artemov. V.G.; Volkov. A.A.; Sysoev. N.N.; Volkov. A.A. Conductivity of aqueous HCl. NaOH and NaCl solutions: is water just a substrate? *EPL* **2015**, 109. 26002.

[S4] Lileev. A.S.; Loginova. D.V.; Lyashchenko. A.K. Dielectric properties of aqueous hydrochloric acid solutions. *Mendeleev Commun.* **2007**, 17. 364-365.

[S5] Catenaccio. A.; Daruich. Y.; Magallanes. C. Temperature dependence of the permittivity of water. *Chem. Phys Lett.* **2003**, 367. 669-671.

[S6] Partanen. J. I.; Juusola. P. M.; Vahteristo. K. P.; de Mendonça. A. J. G. Re-evaluation of the activity coefficients of aqueous hydrochloric acid solutions up to a molality of 16.0

mol·kg^{-1} using the Hückel and Pitzer equations at temperature from 0 to 50 °C. *J. Sol. Chem*. **2007**, *36*. 39-59.

[S7] Saluja. P. P. S.; Pitzer. K. S.; Phutela. R. C. High-temperature thermodynamic properties of several 1:1 electrolytes. *Can. J. Chem*. **1986**, *64.* 1328-1335.

[S8] Holmes. H. F.. Simonson. J. M.. Mesner. R. E.. Archer. D. G.. Wood. R. H. The enthalpy of dilution of HCl(aq) to 648 K and 40 MPa. Thermodynamic properties. *J. Chem. Thermodyn.* **1987**, *19*. 863-890.
